# The Role of Self-Efficacy and Risk Perception in the Willingness to Respond to Weather Disasters Among Emergency Medicine Health Care Workers in Pakistan

**DOI:** 10.1017/dmp.2023.126

**Published:** 2023-07-21

**Authors:** Bee-Ah Kang, Daniel J. Barnett, Ume-e-Aiman Chhipa, Amber Mehmood, Badar Afzal, Junaid Razzak, Nargis Asad

**Affiliations:** 1 Department of Health, Behavior and Society, Johns Hopkins Bloomberg School of Public Health, Baltimore, MD, USA; 2 Department of Environmental Health & Engineering, Johns Hopkins Bloomberg School of Public Health, Baltimore, MD, USA; 3 Centre of Excellence for Trauma and Emergencies, Aga Khan University, Karachi, Pakistan; 4 Department of Public Health, University of South Florida College of Public Health, Tampa, FL, USA; 5 Department of Emergency Medicine, Aga Khan University, Karachi, Pakistan; 6 Department of Emergency Medicine, Weill Cornell Medical College, New York, NY, USA; 7 Department of Psychiatry, Aga Khan University, Karachi, Pakistan

**Keywords:** disaster medicine, self efficacy, willingness to respond, extended parallel process model, emergency health workers

## Abstract

**Objective:**

Optimizing health care workers’ (HCWs) willingness to respond (WTR) is critical in low-and-middle-income countries (LMICs) for proper health system functioning during extreme weather events. Pakistan frequently experiences weather-related disasters, but limited evidence is available to examine HCW willingness. Our study examined the association between WTR and behavioral factors among emergency department HCWs.

**Methods:**

A cross-sectional survey was conducted from August to September 2022 among HCWs from 2 hospitals in Karachi, Pakistan. Non-probability purposive sampling was used to recruit participants. A survey tool was informed by Witte’s Extended Parallel Process Model (EPPM). Multivariate logistic regression analyses were performed to examine the association between WTR and attitudes/beliefs as well as EPPM profiles.

**Results:**

Twenty-nine percent of HCWs indicated a low WTR. HCWs using public transportation had a higher WTR. Perceived knowledge and skills, self-efficacy, and perceived impact of one’s response showed positive associations with WTR if required. Perception that one’s colleagues would report to work positively predicted WTR if asked. Consistent with the EPPM, HCWs with high efficacy and perceived threat were willing to respond to weather disasters.

**Conclusions:**

Our findings highlight the need of strengthening WTR by promoting self-efficacy and enhancing accurate risk perception as a response motivator, among emergency department HCWs in Pakistan.

Climate change-related hazards and extreme weather events have drastically increased in the past 5 decades, with nearly 5 billion people affected globally.^1^ Mounting evidence supports that weather-related disasters, including floods, drought, and rising temperatures have led to a number of health challenges such as respiratory illness, waterborne diseases, mental health problems, and premature deaths.^1–6^ The consequences of weather-related disasters predominantly fall on low-and-middle-income countries (LMICs) due to their geo-climatic surroundings, lack of preparedness in health care, and socioeconomic and political factors.^7–9^

In Pakistan, one of the most disaster-prone countries, the frequency and severity of weather-related disasters have intensified in recent years, causing numerous health issues and harm to its economic development.^10,11^ Pakistan’s mountain ranges contain a large volume of fresh water from snow and glaciers, leading scientists to deem this region under grave threat due to climate change. Most recently, Pakistan’s 2022 floods led to 12 867 individuals being injured, 1739 dead, and 8 million people being displaced.^12,13^ While Pakistan experienced this unprecedented crisis with more than a third of the country underwater affecting 33 million people, its fragile health care system and infrastructure posed additional challenges in emergency response and rehabilitation.^12,14^

Despite the need for adequate health system functioning during weather disasters, little attention has been paid to health care workers (HCWs) and their willingness to report to work in large-scale crisis events in LMIC settings. The willingness to respond^15^ (WTR) is an indispensable element of effective health system functioning in emergencies and disasters. *Willingness* refers to the “attitudinal” dimension of health crisis management, in definitional and practical contrast with *ability*, which comprises knowledge and skills.^15,16^ Deficits in response willingness among HCWs during public health emergencies represent a critical stress-point for local, regional, and global health security. Therefore, strategies to boost response willingness are akin to bolstering a stressed health system.

In LMICs, where gaps in health system resilience and significant additional unknowns are present in the face of resource challenges, any deficits in HCWs’ WTR can inhibit sustainable provision of health services during weather disasters. Particularly, since the emergency department (ED) forms a foundation of the health care system in many LMICs, a shortage of HCWs due to gaps in WTR would adversely affect crisis care in weather-related disasters. The unwillingness to respond is a particularly salient consideration among emergency HCWs in LMICs who play a critical role in and are potentially exposed to a variety of high-stress situations in disaster response.

Nascent literature suggests marked gaps in the willingness of health care workers to respond to public health emergencies and disasters in LMICs, where the work is demanding and salaries are often poor.^17,18^ In Yemen, for example, a 2018 large multihospital-based study of HCWs indicated that 23% conveyed a low WTR to a weather disaster.^19^ A study in Pakistan further highlighted that 20% of medical staff refused to report for duty during the pandemic, and their WTR was influenced by role competence and self-efficacy.^20^ While it is essential to investigate behavioral attributes related to WTR to effectively foster disaster response capacity, there remains little robust evidence. A systematic review indicated that theory-driven approaches have been less used in LMIC settings, particularly in Asian countries that bear greater incidence of disasters with different cultural characteristics than in high-income countries in which theories have been predominantly applied and tested.^21^

In light of the salience of weather disaster response willingness among HCWs and the need to address theoretical gaps, we conducted a survey of emergency medicine department workers in Pakistan to assess the association between multi-dimensional behavioral factors and their WTR across various weather-related emergency contexts. We also gauged the influence of perceived threat and efficacy on WTR in such events based on Witte’s perceived threat- and efficacy-centered Extended Parallel Process Model (EPPM), which supports that risk management behaviors can be promoted through raising risk perception and self-efficacy.^22^

## Methods

### Study Design

We conducted a cross-sectional survey from August 16 to September 10, 2022, among emergency department HCWs in 2 hospitals (Aga Khan University Hospital [AKUH] and Jinnah Postgraduate Medical Centre [JPMC]) located in Karachi, Pakistan. The survey aimed to examine participants’ WTR to weather-related disasters in their own setting and how their willingness was associated with demographic characteristics and behavioral and psychosocial factors, including self-efficacy, risk perception, perceived knowledge, and skills.

### Sample

Non-probability purposive sampling was used to recruit participants. Emergency department HCWs from AKUH and JPMC were invited to participate in the study. Participants were considered eligible to be included in the study if they (1) were age 18 years or older, (2) had worked in their current job position for at least 3 months, and (3) were a current employee in the emergency department at AKUH or JPMC, including clinical staff involved in direct patient care (eg, doctors, nurses, and technicians).

A recruitment letter was disseminated via hospital-wide emails. Interested individuals contacted the study team via email, phone, or WhatsApp. HCWs who agreed to conduct the online survey were provided with a link to the survey by the research team, and they were informed that their survey initiation implied consent. Those who participated in the written (in-person) survey met with a research coordinator and provided oral consent to participate. Among 370 eligible HCWs from the 2 hospitals, we aimed to recruit 250 individuals, accounting for a potential non-response rate. In total, 362 individuals completed the survey. This sample constituted 97.83% of ED staff.

### Data Collection

We collected our data through in-person and online surveys (Qualtrics, Seattle, WA), with identical survey questions implemented for both versions. The survey tool consisted of 2 main parts: a demographic section and an attitudes/beliefs section. In the demographic section, we collected both demographic and professional information, including parental status, household dynamics, primary role in the department, and work hours. The subsequent section focused on HCWs’ attitudes and beliefs toward responding to weather-related disasters along with overall training experiences in emergency preparedness.

The study tool was available in English for the self-administered online survey, and in-person surveys were administered by an interviewer either in Urdu or English based on participants’ language preference. In-person survey responses were recorded by the data collector in a paper-based form. Given heavy work demands in the EDs, the survey was designed to be completed approximately within 20 minutes, with a shorter duration for the online survey. Data were anonymized, stored in a password-protected device, and accessed by the research team only.

Research ethics approvals were received from the Johns Hopkins Bloomberg School of Public Health Institutional Review Board (IRB00019662), the JPMC Institutional Review Board (f.2-81/2022-GEN/133/JPMC), and the AKUH Ethics Review Committee (6959).

### Measurement

The survey questionnaire was adapted from previous research that validated the instrument in other disaster scenarios, including influenza pandemics and radiological bombing events.^23–25^ EPPM-based threat and efficacy measures evolved from prior evidence were tested in multiple countries and health conditions.^26^ We refined the questionnaire to reflect the circumstances relevant to weather-related emergencies and hospital settings in Pakistan.

The questionnaire contained basic demographic questions (eg, age, gender, hospital affiliation, job classification, length of service in the current organization, role in the department). Other key demographic information, including being a single parent, living with older adults or children, and using public transportation to commute, was measured with a binary response option. Self-efficacy was assessed by using the General Self-Efficacy (GSE) scale that shows a good reliability score with alpha coefficients ranging from 0.76 to 0.90,^27^ and it showed high internal consistency of 0.88 in Pakistan.^28^ The 10 items of the GSE scale were included with the response options of a 4-point Likert scale ranging from 1 (“Not at all true”) to 4 (“Exactly true”). The behavioral constructs comprised 27 questions that asked about HCWs’ willingness to report to work during weather-related emergencies and their associated attitudes and beliefs, including perceived occurrence and severity of weather disasters in their regions, perceived knowledge and skills, psychological preparedness, and readiness of hospital response. Responses were measured with a 9-point Likert scale (1 = “Strongly agree”; 9 = “Strongly disagree,” or “Don’t know”). The questionnaire was translated into Urdu by the local research team for in-person facilitation and was pre-tested to ensure an accurate reflection of the original survey.

### Data Analysis

All responses to the attitudes and beliefs statements were dichotomized into categories of positive response (≤ 4) versus negative response (> 4). Also, levels of threat and efficacy were calculated as the product of the participants’ responses to 2 statements on perceived threat (ie, perceived occurrence and perceived severity) and 2 statements on perceived efficacy (ie, perceived ability to perform their duty and perceived impact of their performance). Subsequently, categories of perceived threat and efficacy were dichotomized into “High” and “Low” by the median value of each construct. Based on this assessment, participants were assigned to 1 of 4 EPPM profiles: low threat and low efficacy (LT/LE), low threat and high efficacy (LT/HE), high threat and low efficacy (HT/LE), and high threat and high efficacy (HT/HE).

Univariate logistic regression analyses were performed to identify key demographic and work-related determinants of WTR. Additionally, multivariate logistic regression analyses adjusting for the key demographic characteristics were performed to examine the association between WTR and attitudes/beliefs as well as EPPM profiles. Participants’ prior training experiences in emergency preparedness were assessed by calculating frequencies and percent. All analyses were performed using 2018 STATA version 14.2 for Mac (StataCorp, College Station, TX).

## Results

### Demographic Profile

Responses from 313 ED health workers were analyzed after excluding incomplete data (n = 49) that only had “Don’t know” responses or did not contain any responses in WTR and belief statements (Table 1)—181 (57.83%) participants were females and 132 (42.17%) were males; more than half of the participants (n = 197, 62.94%) were ages 20 to 29; 194 participants had a bachelor’s degree, and approximately one-fifth (n = 64, 20.51%) had a professional degree; 39 (12.54%) participants were single parents, and 124 (39.62%) participants were living with children; more than half (n = 182, 58.15%) were living with older adult dependents; 229 (73.63%) health workers worked over 40 hours per week on average; and most participants were either resident physicians (n = 118, 37.70%) or nurses (n = 118, 37.70%).

### Willingness-to-Respond by Demographics

Associations between demographic characteristics and WTR to weather-related disasters are presented in Table 2. Health workers’ WTR was 71.38% if required and 67.14% if asked. When asked about their WTR if required, those living with pets reported being 54% less likely to be willing to respond than those without pets (odds ratio [OR] 0.46, 95% CI: 0.22–0.96). Health workers using public transportation for commuting showed 2.28 greater odds of WTR if required than those not using public transportation (OR 2.28, 95% CI: 1.30–3.99). Other characteristics, including gender, age, education, and work-related attributes, were not associated with WTR if required.

### Willingness to Respond and Behavioral Factors

Associations between attitudes/beliefs about weather-related disasters and WTR are detailed in Table 3. Several demographic and professional characteristics (ie, education, living with pets, living with children, using public transportation, and work hours) were found to be independently associated with both WTR if required and WTR if asked in a multivariate analysis, and were controlled in subsequent logistic regression analyses.

After adjusting for these factors, most attitudes/beliefs were significantly associated with WTR if required. Health workers who perceived themselves to have skills for role-specific responsibilities showed 36 greater odds of responding to weather disasters than those who did not perceive that they had skills (OR 36.18, 95% CI: 16.20–80.76). Furthermore, perceived knowledge about the public health impact (OR 29.75, 95% CI: 14.08–62.88), perceived importance of one’s role (OR 23.94, 95% CI: 11.32–50.63), psychological preparedness to perform responsibilities (OR 22.15, 95% CI: 10.56–46.49), and perceived need for training (OR 23.20, 95% CI: 11.02–48.84) were significantly associated with WTR if required. Disaster-specific self-efficacy, including perceived ability to perform (OR 11.77, 95% CI: 6.15–22.52) and perceived high impact of one’s response (OR 26.40, 95% CI: 12.76–54.65), showed significant associations with WTR if required. One’s general self-efficacy was not statistically significant.

Similarly, most attitudes/beliefs were significantly associated with WTR if asked, but with lower odds ratios than WTR if required overall. Those who perceived that colleagues would report to work in weather-related disasters were 16 times more likely to be willing to respond than those who did not (OR 16.20, 95% CI: 8.33–31.56). Perceived knowledge about the public health impact (OR 11.50, 95% CI: 6.06–21.84), perceived importance of one’s role (OR 10.44, 95% CI: 5.34–20.42), and perceived need for training (OR 11.46, 95% CI: 5.93–22.13) were significantly associated with WTR if asked. Also, perceived ability to perform (OR 8.76, 95% CI: 4.83–15.89) and perceived impact of one’s response (OR 15.89, 95% CI: 8.28–30.52) were significantly associated with WTR if asked, but general self-efficacy was not significant.

### Willingness to Respond and EPPM Constructs

Approximately 54.95% of participants indicated low perceived threat, and 53.99% indicated low perceived efficacy toward weather disasters (Table 4). After adjusting for key demographic factors, respondents who perceived higher threat toward weather disasters were 14 times more likely to be willing to respond if required than those who had lower perceived threat (OR 14.49, 95% CI: 6.66–31.50), and participants who had higher efficacy were 17 times more likely to be willing to respond if required than their counterparts (OR 17.41, 95% CI: 7.80–38.84). With regard to EPPM-based profiles, approximately one-third of participants (33.23%) were in the high threat/high efficacy profile, whereas 42.17% of participants were in the low threat/low efficacy profile. Health workers in the high threat/high efficacy profile were 35 times more likely to be willing to respond to weather-related disasters than those in the low threat/low efficacy profile (OR 34.63, 95% CI: 12.65–94.81). Consistent with WTR if required, participants with high perceived threat and high efficacy were more likely to be willing to respond to weather-related disasters compared with their counterparts (OR 3.50, 95% CI: 1.99, 6.15; OR 5.98, 95% CI: 3.29–10.86, respectively). Additionally, compared with health workers in the low threat/low efficacy profile, those in the high threat/high efficacy profile (OR 7.63, 95% CI: 3.77–15.44) were significantly more likely to be willing to respond to weather disasters.

### Training Experience

Participants’ emergency preparedness training experiences were assessed (Table 5). Among 278 health workers who responded to the training-related questions, 79 (28.42%) reported not receiving any prior emergency preparedness training. The most common form of prior preparedness training occurred face to face (33.09%), followed by academic coursework (30.21%), and full-scale drills or exercises (26.62%). Only 10.07% and 9.35% of health workers reported having tabletop exercises and real-life disaster experience, respectively.

## Discussion

Our study examined the associations between demographic and professional characteristics and WTR and assessed the relationships between behavioral factors and WTR based on the EPPM. Perceived knowledge and skills, perceived importance of one’s role, disaster-specific self-efficacy, and response efficacy (perceived high impact of one’s response) showed particularly strong associations with WTR if required. Perceived likelihood of colleagues’ reporting to work, along with self-efficacy, had significant positive associations with WTR if asked. Consistent with the EPPM theory, individuals with high threat and efficacy perception were most likely to be willing to respond to weather disasters.

Our findings of 71% of HCWs showing willingness to respond are consistent with previous studies in LMICs; 77% of HCWs in Yemen expressed a high willingness to respond to weather disasters,^19^ and 72% of nurses in Iran reported being willing to respond to earthquakes.^29^ Evidence on health care personnel response willingness varies across disaster scenarios. The same studies found that only 66%^19^ and 55%,^29^ respectively, of health workers were likely to report during pandemic emergencies, and other scenario comparison studies in high-income settings also demonstrated health workers’ lower willingness to report to work during an influenza pandemic compared with weather crises.^30,31^ Weather disasters seem to induce less fear of contagiousness and uncertainties about consequences, creating less burden on work attendance. Nevertheless, relatively high WTR among our participants does not guarantee proper health system functioning given Pakistan’s growing vulnerabilities toward climate change-driven hazards that are coupled with unique geographical and sociopolitical conditions as well as its already-drained health system capacity after the COVID-19 pandemic.^20,32^ This is specifically notable that the HCWs who participated in the study were not situated directly in the flood-affected areas, which may have some bearing on risk perception. The need to understand WTR and its context-specific attributes is imperative to provide the workforce with proper support.

Our results highlight the role of perceived skills and knowledge in promoting WTR through role-specific training. A study conducted in Malaysia also revealed that the level of skills and knowledge was significantly associated with disaster preparedness among nurses,^33^ and another study in the United States suggested that having a specified role in the workplace plan increased the likelihood of nurses responding to a disaster.^34^ While less is known about the type and extent of training that provides sufficient qualifications for HCWs, particularly in the weather disaster context, some evidence indicated that employing various training modalities is effective given hospital demands and staff shortages.^35–37^ Our participants’ hospitals in Pakistan do not currently offer formal disaster response training beyond certain lecture modules, as also evinced by our findings that few HCWs were exposed to opportunities that reflect real-life disaster scenarios and practical demonstration of skills, especially in those situations where the health facility itself could be directly affected by the disaster. Therefore, we recommend implementing training programs tailored to participants’ specific roles and sociodemographic characteristics, such as educational background, using various platforms to further engage HCWs. Given prior evidence that an actual disaster experience may increase WTR and disaster preparedness,^34,37^ we also encourage hospital leadership and stakeholders to gather evidence from Pakistani health care staff preparedness and response to recent floods to further inform training programs.

Our EPPM-based findings point to the need for strengthening self-efficacy and perceived threat through training as a key to boosting HCWs’ response willingness. We have found that those who fit a highly confident and concerned profile are significantly more likely to report for duty than those less concerned and confident. This is consistent with prior evidence that educational materials for HCWs need to highlight the significance of an event and their designated roles, not only to instill a sense of urgency to motivate them to take action, but also to make them feel confident in their ability (self-efficacy) and believe their work will lead to desirable outcomes (response efficacy).^23,24^ While a sizable body of research has focused on the role of general self-efficacy in work competence,^38,39^ our findings implied that fostering disaster- and role-specific self-efficacy may be more effective in fostering HCW motivation to report in support of other existing evidence.^19,40^ It is thus critical to regularly offer role-specific training for HCWs up to a point at which they feel confident in performing their responsibilities during real-life disaster events, delivering messages that a weather disaster is a viable threat along with scientific evidence on the impact of HCWs’ performance. According to Bandura,^41^ self-efficacy is enhanced through mastery performance, vicarious experience (modeling), verbal persuasion, and physiological arousal (anxiety); utilizing these strategies in training can be particularly useful for the workforce.

Consistent with other research on willingness in disaster settings,^23–25^ we found a lower WTR if asked and its weaker associations with attitudes and beliefs, compared with WTR if required. It is reasonable to expect from this finding that HCWs are at risk for “voluntary absenteeism” when instructions from their organizations are void during emergencies. Among attitude/belief constructs, perception about colleagues’ likelihood of reporting showed particularly strong associations with WTR if asked. Our data further validate this finding since participants’ willingness was higher when they believed their peers were also responding, even when compared to willingness if additional compensation was available to them. HCWs’ perceptions that colleagues would attend to work during emergencies may serve as “social nudges,” accelerating their voluntary report to duty. The social norms theory explicates that people’s behaviors are likely to change based on their perception about the frequency of others’ behaviors (descriptive norms) and beliefs about what others expect them to do (injunctive norms).^42^ Although these theoretical mechanisms of norms have been less explored for disaster response personnel, our findings contributed to practical implications for hospital leadership and policy makers, calling for communication approaches that emphasize the collaborative nature of disaster management to create a sense of collective identity among the disaster response workforce within a hospital.

Furthermore, our study points to the need to mitigate barriers that affect HCWs’ ability to report to duty. The availability of transportation was significantly associated with WTR, similar with studies conducted in Israel and the United States.^16,43,44^ In Pakistan, urban roads and highways are prone to flash flooding throughout the monsoon season,^45^ and public transportation services are mostly unavailable during the heavy rainfall. The absence of transportation during weather disasters is particularly perceived as a hindrance among HCWs who rely on public transportation to commute. We accordingly suggest that institutions ensure proper transportation aid to prevent hospitals from facing staffing shortage issues during weather events. Evidence from the COVID-19 pandemic showed that travel allowances for staff and coordination between the health and transport sector to reduce costs for transportation may facilitate the engagement of health workers in LMICs.^46,47^ Studies in the United Kingdom and the United States further argued that interventions and policies that ensure transportation improve HCW willingness.^48,49^ Additionally, our results showed that HCWs living with pets were less likely to report, as also represented in previous work.^16,50^ However, we did not find the effect of other personal responsibilities (ie, child care, older adult care), in contrast with research that identified family concerns as major determinants of WTR.^44,51,52^ This may be due to the prevalent extended-family societal structure, which provides support to HCWs’ caregiving role, and weather disasters induce less fear of infectious disease contagion, a dominant impediment to disaster response in pandemics. Yet, our findings point toward the need to provide HCWs with adequate resources and practical guidelines to address these observed personal challenges, in addition to promoting individual behaviors. Proper disaster preparedness and management plans that outline expectations and rights of health personnel may enhance their work competence and willingness, contributing to the overall functioning and resilience of health systems.^53–55^ Therefore, we urge employers to establish evidence-informed plans that delineate what responsibilities HCWs need to fulfill, what resources and services are offered (eg, family care, transportation aid), and how their rights will be protected (eg, workplace safety) during a crisis.

### Limitations

There are a few limitations in our study. While our overall survey response rate of 97.83% was high, there was rather unequal representation of staff between the 2 hospitals (98.81% of AKUH and 63.27% of JPMC staff). Staff in public hospitals in Pakistan (eg, JPMC) often face staff shortage issues and resultant heavy workloads, which could have decreased our participants’ capacity to participate in our study. We recommend future studies to employ strategies for retaining public hospital staff and expand to other areas in Pakistan to ensure generalizability. Further, we acknowledge that participants’ self-reported WTR and associated behavioral factors may not predict actual behavior. Yet, our theoretical reasoning is supported by Bandura’s studies that suggested that one’s stated self-efficacy is predictive of real-world behavior.^41,56^ Lastly, our study was performed during and shortly after 2022 floods in Pakistan; some HCWs working at the hospitals may not have been available to participate. Our approaches to using both written surveys at the worksite and online surveys, which could be taken remotely, partly addressed this issue. Despite the limitations, our study provided practical guidance on system strengthening in Pakistan, exploring underlying behavioral factors of key health personnel in weather disaster response. Our findings suggested bottlenecks of the health system, which may be applicable to other similar LMICs, including Syria, Bangladesh, and India, that are experiencing impending threats of climate change and its dire consequences.

## Conclusions

In the face of climate change and weather-related disasters, strategies must be undertaken to enhance system resilience and preparedness to ameliorate negative consequences. We recommend implementing hospital-level communication and training programs tailored to fostering efficacy and threat among health workers in limited-resource settings. Additionally, ensuring transportation needs during a crisis, establishing varying training modalities, and strengthening collegiality within the hospital may further boost disaster response willingness among HCWs. Our study provided critical and timely implications for improving WTR in one of the countries with the highest risks for climate change-related disasters. To the best of our knowledge, this is the first theory-driven study to explore the patterns of behavioral factors associated with WTR in the weather disaster context among emergency department HCWs in Pakistan.

## Supplementary Material

Supplemental File 1

Supplemental File 2

## Figures and Tables

**Table 1.** Demographic characteristics of emergency department health workers in Karachi, Pakistan (N = 313)

Sociodemographic characteristics	N	%
Gender		
Male	132	42.17
Female	181	57.83
Age		
20–29	197	62.94
30–39	82	26.20
40–49	24	7.67
50 or older	10	3.20
Education		
High school diploma	35	11.22
Bachelor’s degree	194	62.18
Master’s degree	19	6.09
Professional degree	64	20.51
Single parent		
No	272	87.46
Yes	39	12.54
Living with children		
No	189	60.38
Yes	124	39.62
Living with older adult		
No	131	41.85
Yes	182	58.15
Living with pets		
No	274	87.82
Yes	38	12.18
Using public transportation for commute		
No	191	61.22
Yes	121	38.78
Professional characteristics	N	%
Hospital affiliation		
Aga Khan University	229	75.08
Jinnah Postgraduate Medical College	76	24.92
Primary affiliation		
No	22	7.05
Yes	290	92.95
Length of hospital affiliation		
Less than 1 year	69	22.19
1–5 years	165	53.05
6–10 years	46	14.79
More than 10 years	31	9.97
Work hours per week		
Less than 10 hours	21	6.75
11–19 hours	16	5.14
20–29 hours	5	1.61
30–39 hours	40	12.86
40–49 hours	113	36.33
More than 50 hours	116	37.30
Role in department		
Faculty	14	4.47
Resident physician/fellow	118	37.70
Physician extender (PA; NP)	7	2.24
Nurse	118	37.70
Medical/nursing student	1	0.32
Administration/management	2	0.64
Clinical support staff	18	5.75
Research	3	0.96
Other	32	10.22
Length of role affiliation		
Less than 1 year	75	23.96
1–5 years	167	53.35
6–10 years	43	13.74
More than 10 years	28	8.95

**Table 2.** Associations between participant demographics and willingness to respond to a weather-related disaster (N = 313)

	WTR, if required		WTR, if asked	
	% Agree^a^	OR (95% CI)^b,c^	% Agree^a^	OR (95% CI)^b,c^
**All** ^d^	71.38		67.14	
**Sociodemoeraphic characteristics**				
Gender				
Male	67.21	–	62.60	–
Female	74.40	1.42 (0.85, 2.37)	70.70	1.44 (0.87, 2.38)
Age				
20–29	74.05	–	69.49	–
30–39	64.47	0.64 (0.36, 1.13)	58.67	0.62 (0.36, 1.09)
40–49	63.16	0.60 (0.22, 1.61)	72.22	1.14 (0.39, 3.36)
50 or older	88.89	2.80 (0.34, 22.99)	77.78	1.54 (0.31, 7.64)
Education				
High school	81.25	–	65.62	–
Bachelor’s degree	69.02	0.51 (0.20, 1.32)	67.61	1.09 (0.49, 2.42)
Master’s degree	57.14	0.31 (0.08, 1.22)	57.14	0.70 (0.19, 2.53)
Professional degree	77.97	0.82 (0.28, 2.41)	68.97	1.16 (0.47, 2.91)
Single parent				
No	71.15	–	67.35	–
Yes	71.43	1.01 (0.46, 2.22)	69.70	1.12 (0.51, 2.46)
Living with children				
No	73.14	–	71.69	–
Yes	68.70	0.81 (0.48, 1.35)	60.53	0.61 (0.37, 1.00)
Living with older adult				
No	71.31	–	69.23	–
Yes	71.43	1.01 (0.60, 1.68)	65.64	0.85 (0.51, 1.41)
Living with pets				
No	73.33	–	68.98	–
Yes	55.88	0.46* (0.22, 0.96)	55.88	0.57 (0.28, 1.18)
Using public transportation for commute				
No	64.94	–	65.87	–
Yes	80.87	2.28** (1.30, 3.99)	68.75	1.14 (0.68, 1.90)
**Professional characteristics**				
Hospital affiliation				
Aga Khan University	68.57	–	65.52	–
Jinnah Postgraduate Medical College	80.56	1.90 (1.00, 3.65)	72.46	1.39 (0.76, 2.53)
Primary affiliation				
No	66.67	–	60.00	–
Yes	71.59	1.26 (0.46, 3.48)	67.42	1.38 (0.48, 4.00)
Length of hospital affiliation				
Less than 1 year	77.42	–	65.00	–
1–5 years	69.08	0.65 (0.33, 1.30)	69.86	1.25 (.660, 2.361)
6–10 years	72.73	0.78 (0.32, 1.90)	63.64	0.94 (0.42, 2.12)
More than 10 years	66.67	0.58 (0.22, 1.53)	60.71	0.83 (0.33, 2.10)
Work hours per week				
Less than 10 hours	57.14	–	55.00	–
11–19 hours	71.43	1.88 (0.44, 7.96)	64.29	1.47 (0.36, 6.00)
20–29 hours	75.00	2.25 (0.20, 25.37)	75.00	2.46 (0.22, 27.84)
30–39 hours	77.14	2.53 (0.79, 8.16)	51.52	0.87 (0.29, 2.65)
40–49 hours	75.93	2.37 (0.90, 6.24)	72.64	2.17 (0.82, 5.78)
More than 50 hours	66.98	1.52 (0.59, 3.95)	68.32	1.76 (0.67, 4.68)
Role in department				
Faculty	69.23	–	45.15	–
Resident/fellow	71.82	1.13 (0.33, 3.95)	67.59	2.43 (0.76, 7.78)
Physician extender	71.43	1.11 (0.15, 8.37)	80.00	4.67 (0.40, 53.95)
Nurse	65.14	0.83 (0.24, 2.88)	64.08	2.08 (0.65, 6.65)
Clinical support staff	88.89	3.56 (0.54, 23.39)	70.59	2.80 (0.62, 12.66)
Research	66.67	0.89 (0.06, 12.89)	33.33	0.58 (0.04, 8.15)
Other	81.48	1.96 (0.43, 9.00)	82.14	5.37* (1.25, 23.05)
Length of role affiliation				
Less than 1 year	73.91	–	62.12	–
1–5 years	71.15	0.87 (0.46, 1.65)	69.33	1.38 (0.75, 2.53)
6–10 years	72.50	0.93 (0.39, 2.24)	65.86	1.18 (0.52, 2.66)
More than 10 years	64.00	0.63 (0.24, 1.67)	69.57	1.39 (0.50, 3.86)

a Percent agreeing with WTR statement.

b Odds ratios represent the odds of stating a positive WTR for the respective positive attitude/belief response compared to the negative response.

c *P < 0.05, **P < 0.01, ***P < 0.001.

d Percent pertaining to all survey respondents.

**Table 3.** Associations between attitudes/beliefs and self-reported willingness to respond to a weather-related disaster (N = 313)

Attitude and belief statements	WTR, if required		WTR, if asked	
	% Agree^a^	OR (95% CI)^b,c^	% Agree^a^	OR (95% CI)^b,c^
Perceived likelihood of occurrence in this region	89.19	22.72 (10.85, 47.55)	78.88	5.19 (2.91, 9.27)
Perceived severity of health consequences	87.18	16.39 (8.26, 32.49)	78.42	6.05 (3.34, 10.97)
Perceived likelihood of being asked to report to duty	91.37	37.31 (17.45, 79.76)	81.05	9.65 (5.18, 17.98)
Perceived likelihood that colleagues will report	88.64	14.48 (7.36, 28.46)	86.29	16.20 (8.33, 31.56)
Perceived knowledge about the public health impact	89.95	29.75 (14.08, 62.88)	81.96	11.50 (6.06, 21.84)
Perceived awareness of role-specific responsibilities	88.06	14.57 (7.57, 28.00)	80.51	7.06 (3.88, 12.85)
Perceived skills for role-specific responsibilities	90.40	36.18 (16.20, 80.76)	81.68	8.22 (4.54, 14.85)
Perceived importance of one’s role in the hospital’s response	87.25	23.94 (11.32, 50.63)	79.00	10.44 (5.34, 20.42)
Psychological preparedness	87.86	22.15 (10.56, 46.49)	79.40	7.41 (4.03, 13.61)
Perceived confidence in safety to get to work	84.30	6.58 (3.53, 12.28)	78.24	4.04 (2.32, 7.04)
Perceived confidence in personal safety at work	84.70	9.55 (4.88, 18.69)	82.02	9.22 (4.95, 17.19)
Perceived preparedness of family in absence	88.96	12.63 (6.46, 24.70)	81.53	5.34 (3.01, 9.47)
Perceived hospital ability to provide timely information	87.37	14.15 (7.36, 27.17)	80.98	8.09 (4.46, 14.70)
Perceived need for pre-event preparation and training	86.89	23.20 (11.02, 48.84)	80.60	11.46 (5.93, 22.13)
Perceived need for during/post-event psychological support	87.10	11.29 (5.99, 21.29)	81.56	7.51 (4.16, 13.57)
**Self-efficacy and response efficacy**				
General self-efficacy	76.54	1.29 (0.78, 2.16)	70.62	1.49 (0.89, 2.48)
Emergency-related self-efficacy				
Perceived ability to perform duties	86.63	11.77 (6.15, 22.52)	82.42	8.76 (4.83, 15.89)
Perceived ability to address patient concerns	88.72	20.04 (10.03, 40.04)	81.58	9.53 (5.16, 17.59)
Perceived high impact of one’s response	90.16	26.40 (12.76, 54.65)	85.11	15.89 (8.28, 30.52)

a Percent agreeing with WTR statement.

b Odds ratios represent the odds of stating a positive WTR for the respective positive attitude/belief response compared to the negative response.

c All statistically significant associations showed a significance of *P* < 0.001.

**Table 4.** Associations between EPPM categories and self-reported willingness to respond to a weather-related disaster (N = 313)

Extended Parallel Process Model profile	n (%)^a^	WTR, if required		WTR, if asked	
		% Agree^b^	OR (95% CI)^c,d^	% Agree^b^	OR (95% CI)^c,d^
Low threat	172 (54.95)	51.63	–	54.42	–
High threat	141 (45.04)	93.43	14.49 (6.66, 31.50)	81.20	3.50 (1.99, 6.15)
Low efficacy	169 (53.99)	48.98	–	48.57	–
High efficacy	144 (46.00)	94.41	17.41 (7.80, 38.84)	85.71	5.98 (3.29, 10.86)
Low threat/low efficacy	132 (42.17)	37.17	–	43.52	–
Low threat/high efficacy	40 (12.78)	92.50	19.64 (5.55, 69.50)	84.62	6.34 (2.41, 16.67)
High threat/low efficacy	37 (11.82)	88.24	13.61 (4.33, 42.77)	65.62	2.35 (1.01, 5.47)
High threat/high efficacy	104 (33.23)	95.15	34.63 (12.65, 94.81)	86.14	7.63 (3.77, 15.44)

a Frequencies and percent of respondents in each respective threat and efficacy category.

b Percent agreeing with WTR statement.

c Odds ratios represent the odds of stating a positive WTR for the respective positive attitude/belief response compared to the negative response.

d All statistically significant associations showed a significance of *P* < 0.001.

**Table 5.** Disaster preparedness training experiences among emergency department health workers in Karachi, Pakistan (N = 278)

Training/disaster experience		N	%
Any training	None	79	28.42
	Some	199	71.58
Tabletop exercise	No	250	89.92
	Yes	28	10.07
Full-scale drill/exercise	No	204	73.38
	Yes	74	26.62
Academic coursework	No	194	69.78
	Yes	84	30.21
Face-to-face training/lecture	No	186	66.89
	Yes	92	33.09
Writing emergency/disaster management plans	No	240	86.33
	Yes	38	13.66
Real-life disaster experience	No	252	90.65
	Yes	26	9.35

## Abbreviations:

AKUH: Aga Khan University Hospital
ED: emergency department
EPPM: Extended Parallel Process Model
GSE: general self-efficacy
HCW: health care worker
JPMC: Jinnah Postgraduate Medical Centre
LMIC: low-and-middle-income country
WTR: willingness to respond
