# Supplementary material for: The Role of Self-Efficacy and Risk Perception in the Willingness to Respond to Weather Disasters Among Emergency Medicine Health Care Workers in Pakistan
Source: Disaster Med Public Health Prep. Author manuscript; Available in PMC 2024 May 20. (PMC11103185; doi:10.1017/dmp.2023.126)
Supplement: Supplemental File 1 [file NIHMS1990791-supplement-Supplemental_File_1.docx]

**Supplement 1. Recruitment Letter for Emergency Department Health Workers**

Dear [insert name],
We are researchers at Aga Khan University, Weill Cornell Department of Emergency Medicine, University of South Florida College of Public Health and Johns Hopkins University. We writing to invite you to participate in a short (15–20-minute survey) as part of a research study on the willingness of health workers to respond to disasters in the workplace. We hope to use the results of this study to make better trainings and support for health workers like yourself to feel more prepared for disaster scenarios.

We are contacting you because you are a current employee of the Emergency Department or clinical staff involved in direct patient care (doctors, nurses, and technicians) at either Aga Khan University or Jinnah Postgraduate Medical Centre. Participants of any gender, race, and ethnicity are eligible to participate, but you must be 18 years of age or older and in your current position for at least three months.

Your participation in this study, as well as your survey responses, will not be shared with your employers. Participation, or lack thereof, in this study will not impact your employment.

While there is no direct benefit to you from participating in this survey, we will use the results to help people like yourself to have access to better training and support for responding to disaster situations in your workplace.

This project is funded by a National Institutes of Health grant.

Remember, this is completely voluntary. You can choose to be in the study or not. If you'd like to participate or have any questions about the study, we please contact the Principal Investigator, Dan Barnett, MD at [dbarnet4@jhu.edu](mailto:dbarnet4@jhu.edu) or +1-410-502-0591. You may also contact the local team lead, Dr. Nargis Asad, at [nargis.asad@aku.edu](mailto:nargis.asad@aku.edu) or +92 21 34864691.

Thank you very much.

Sincerely,
Daniel J. Barnett, MD, MPH

Associate Professor

Johns Hopkins Bloomberg School of Public Health

Department of Environmental Health and Engineering

615 N. Wolfe Street, Room E7036

Baltimore, Maryland 21205

Phone: 410-502-0591

Fax: 410-955-0617

Email: [dbarnet4@jhu.edu](mailto:dbarnet4@jhu.edu)
