# Supplementary material for: The Role of Self-Efficacy and Risk Perception in the Willingness to Respond to Weather Disasters Among Emergency Medicine Health Care Workers in Pakistan
Source: Disaster Med Public Health Prep. Author manuscript; Available in PMC 2024 May 20. (PMC11103185; doi:10.1017/dmp.2023.126)
Supplement: Supplemental File 2 [file NIHMS1990791-supplement-Supplemental_File_2.docx]

**Supplement 2. Survey Instrument**

Below you will find a set of demographic questions relating to your role at the hospital. Please answer these questions as thoroughly and accurately as possible. Please complete the following information about your personal characteristics

1. With which hospital are you affiliated?
2. How long have you worked at this hospital?
   1. Less than 1 year
   2. 1-5 years
   3. 6-10 years
   4. More than 10 years
3. How many hours per week do you spend working at this hospital?
   1. Less than 10 hours
   2. 11-19 hours
   3. 20-29 hours
   4. 30-39 hours
   5. 40-49 hours
   6. 50 + hours
4. Is this hospital your PRIMARY place of employment?
   1. Yes
   2. No
5. What is your primary role in your department?
   1. Faculty
   2. Resident physician/Fellow
   3. Physician Extender (PA; NP)
   4. Nurse
   5. Medical/Nursing student
   6. Senior Executive
   7. Administration/Management
   8. Clinical Support Staff (Nursing aides; Support technicians; Respiratory/Physical therapists)
   9. Clerical Staff
   10. Maintenance Staff (Facilities; Supply Chain staff)
   11. General Services Employee (Food Services staff; Environmental Services staff)
   12. Information Technology
   13. Finance
   14. Laboratory
   15. Pharmacy
   16. Radiology
   17. Research
   18. Safety Officers
   19. Security
   20. Social Work/Counselors
   21. Other (please specify)
6. How long have you worked in this role?
   1. Less than 1 year
   2. 1-5 years
   3. 6-10 years
   4. More than 10 years
7. What is the highest education level you have completed?
   1. Professional Degree (MD, PhD, PharmD, etc.)
   2. Master's Degree
   3. Bachelor's Degree
   4. High School Diploma/GED
8. What is your gender?
   1. Male
   2. Female
9. What is your age?
   1. Under 20
   2. 20-29
   3. 30-39
   4. 40-49
   5. 50-59
   6. 60 or older
10. Do you rely on public transportation to get to/from work?
    1. Yes
    2. No
11. Do you have any elder family members living with/near you who rely on you for care and/or support?
    1. Yes
    2. No
12. Do you have any children living with you?
    1. Yes
    2. No
13. Are you a single parent?
    1. Yes
    2. No
14. Do you have any pets that rely solely on you for daily care?
    1. Yes
    2. No

We would like to ask your opinion about an emergency scenario related to weather-related emergencies (e.g.floods, drought, and heatwaves). Your responses will be scaled from 1 to 9, where 1 = Strongly agree, and 9 = Strongly disagree. If you are unsure of your response, you may also select, ‘Don’t know’.

1. A weather-related emergency is likely to occur in this region.
2. If it occurs, a weather- related emergency in this region is likely to have severe health consequences.
3. I may be asked to report to duty by the hospital if a weather-related emergency occurs.
4. If I were required by my hospital to report to duty in a weather-related emergency, I would report.
5. If I were asked, but not required, by my hospital to report to duty in a weather- related emergency, I would report.
6. My colleagues/co-workers are likely to report in the event of a weather-related emergency.
7. I am knowledgeable about the potential medical impacts of a weather- related emergency.
8. I know what my role-specific responsibilities are in the event of a weather-related emergency.
9. In terms of my skills, I am prepared to perform my role-specific responsibilities in the event of a weather- related emergency.
10. My role in the hospital's overall response to a weather-related emergency is important.
11. I am psychologically prepared to perform my role-specific responsibilities in the event of a weather-related emergency.
12. I am confident that I could safely get to work to perform my emergency response duties during a weather-related emergency.
13. I am confident I would be safe in my work during response to a weather- related emergency.
14. I would be able to perform my duties successfully in the event of a weather- related emergency.
15. My family is prepared to function in my absence if am called into duty for a weather-related emergency
16. I would be willing to perform my duties during a weather-related emergency even if I was required to work longer than scheduled hours
17. The hospital will be able to provide timely updates about the developing situation during a weather- related emergency
18. I would be able to address the questions of a concerned patient during a weather-related emergency
19. The hospital should provide pre-event preparation and training for weather-related emergencies
20. It is important for me to have some form of psychological support or counseling available during or after a weather-related emergency
21. If I perform my role successfully it will make a difference in the success of the response to a weather- related emergency
22. I would be willing to respond to a weather-related emergency regardless of its severity
23. I would be willing to respond to a weather-related emergency if I knew my colleagues/co-workers were also going to respond
24. I would be willing to respond to a weather- related emergency if a vaccine/daily preventive medications were made available to me
25. I would be willing to respond to a weather- related emergency even if a vaccine/daily preventive medications were NOT available or provided for all staff
26. I would be willing to respond to a weather- related emergency if it was a specific condition of my continued employment
27. I would be willing to respond to a weather- related emergency if there was additional compensation for doing so

Please write your responses below:

1. Please list the most significant factors that would assist you in fulfilling your job requirements during a weather-related emergency
2. Please list the most significant barriers or problems that would prevent you from fulfilling your job requirements during a weather-related emergency

To what extend do you agree with the following statements? Your responses will be scaled from 1 to 4, where 1 = Not at all true, 2 = Hardly true, 3 = Moderately true, and 4 = Exactly true.

1. I can always manage to solve difficult problems if I try hard enough.
2. If someone opposes me, I can find the means and ways to get what I want.
3. It is easy for me to stick to my aims and accomplish my goals.
4. I am confident that I could deal efficiently with unexpected events.
5. Thanks to my resourcefulness, I know how to handle unforeseen situations.
6. I can solve most problems if I invest the necessary effort.
7. I can remain calm when facing difficulties because I can rely on my coping abilities.
8. When I am confronted with a problem, I can usually find several solutions.
9. If I am in trouble, I can usually think of a solution.
10. I can usually handle whatever comes my way.

Please indicate any emergency preparedness training you have received and/or any actual disaster planning/response experience you may have.

- 1. None
  2. Tabletop Exercise(s)
  3. Full-Scale Drill(s)/Exercise(s)
  4. Academic Coursework
  5. Face-to-Face training(s)/lecture(s)/presentation(s)
  6. Writing Emergency Management Plans
  7. Real Life Disaster Experience, please explain_________
